# Effect of distance learning on the quality of life, anxiety and stress levels of dental students during the COVID-19 pandemic

**DOI:** 10.1186/s12909-022-03382-y

**Published:** 2022-04-23

**Authors:** Zeynep Başağaoğlu Demirekin, Muhammed Hilmi Buyukcavus

**Affiliations:** 1 Department of Prosthodontics, Faculty of Dentistry, Suleyman Demirel University, Isparta, Turkey; 2 Department of Orthodontics, Faculty of Dentistry, Suleyman Demirel University, Isparta, Turkey

**Keywords:** Quality of life, DASS-21 scale, Dental students, COVID -19

## Abstract

**Background:**

The long-term psychological effects of COVID-19 on dental students are unclear. The aim of this cross-sectional online study was to investigate the psychological effects of the COVID-19 pandemic on dental students.

**Method:**

The Quality of Life Scale (WHOQOL-BREF) was sent to all dental students through Google Forms to evaluate their quality of life (QoL), and the DASS-21 scale was used to evaluate their psychosocial status due to distance learning during the COVID-19 pandemic. The answers were analyzed both on the basis of year of education and type of education (online versus classroom learning). One-way ANOVA was used for comparison of students in the different years of education; post hoc LSD test was used for pairwise comparisons. Sample t-test was used to compare the two groups separated as classroom/face-to-face learning and distance/online learning.

**Result:**

The questionnaire was completed by 580 students with a response rate of 87.74%. According to the QoL scale results, there was no significant difference between the groups regarding general health, physical health, and psychology, both between different years and learning methods (*p* > 0.05). According to the results of the DASS-21 scale, anxiety and depression in the 3rd year students were significantly higher than the other years. The stress level of the 2nd year students was statistically significantly different from the other years (*p* < 0.05). Evaluation of anxiety, stress and the QoL showed an overall detrimental effect of distance learning on the dental students, although the evaluation did not reach statistical significance.

**Conclusion:**

Anxiety, stress and factors affecting the quality of life negatively affected dental students who received online/distance learning, although the difference did not reach statistical significance when compared to students who received in-classroom learning.

## Introduction

The ongoing COVID-19 pandemic has psychologically affected every layer of society, including dental students who are young and have been left in a state of indefinite uncertainty about the future of their profession [1]. During the COVID-19 pandemic, valuable and practical guidelines provided by the World Health Organization (WHO) were implemented to reduce social contact between individuals, resulting in many educational institutions to temporarily switch to online learning. Thus, the education of dental students continued, and the resulting anxiety was slightly reduced [1–4]. While the COVID-19 pandemic has forced societies to acclimatize to the new circumstances quickly, the education system around the world has also had to adapt. Dental institutions have also had to meet these challenges. Within the scope of the measures taken in the COVID-19 pandemic, dental faculties in Turkey and all over the world started to close as of March 2020, with the aim to minimize contact between students. This was a reflection of successful interventions witnessed in the past to contain and effectively reduce the spread of disease [5–7].

The classical dental education system relies on face-to-face instructor-student relationships and live demonstrations in preclinical laboratories where practical training is prioritized [8]. Due to the implementation of social distancing during the COVID-19 pandemic, greater use of technology became necessary in dental education [9]. Distance e-learning was used as a new teaching method to ensure the progression of dentistry education due to the pandemic related closure of educational institutions [10].

Studies suggest that practical and theoretical equipment used in dentistry education result in a greater level of stress among dentistry students compared to the general population [11]. Although dental education is similar to other natural sciences, there are many points that differ from them. In addition to traditional theoretical and practical medical education, dental education includes materials science and training in the manipulation of these materials. [12]. During these practical trainings, there were many risks in dental education even before COVID-19. During the operation of high-speed and ultrasonic dental instruments, aerosols of fine tissue particles, water, saliva and/or blood are produced. These particles accumulating on surfaces can be a source of indirect contact infection, especially in the respiratory tract [13].Considering this particular risk to dental students, it was thought that the COVID-19 pandemic would have the effect of complicating the working conditions and increasing anxiety and stress for dental students. Despite strong indications of a close relationship between these psychological parameters, anxiety, depression, and stress have not been explored very often in the psychological analysis of dental students.

Scales such as the Dental Environmental Stress Scale (DES), which has been widely used in previous studies, are designed to determine only the level of stress. The Depression, Anxiety and Stress Scale (DASS-21), which has been very popular in recent studies, enables the evaluation of all three dimensions of these psychological states in a single, short and comprehensive scale. Although various studies have evaluated the stress levels and related factors in the dental environment in Turkey, none of the published studies explicitly uses depression and anxiety measures to evaluate psychosocial status and Quality of Life (QoL) of dental students [14, 15].

The COVID-19 pandemic has had adverse effects on students' mental health and education, along with great uncertainty for dental students. Therefore, we aimed to investigate the psychological effects of the COVID-19 pandemic on students during years 1–5 of dental education in a cross-sectional online study.

## Material and methods

### Study type, participants, and sampling procedures

This questionnaire study was carried out between May – June, 2021. Study participants were all undergraduate students of the dentistry program at the Faculty of Dentistry Suleyman Demirel University. The students were strongly encouraged to fill out the questionnaire, but their participation remained voluntary.

Ethical permission for the study was obtained by the Ethics Committee (Decision No: E-87432956–050.99–38980), and the study was carried out in accordance with the Declaration of Helsinki. The students were informed about the study, and the statement that they accepted the study was added to the questionnaire. Although the minimum number of participants to be included in the study was 549 with 90% reliability level and 3% margin of error, more students were included in the study to ensure adequate statistical power. The sample size was calculated using the G*Power software (Franz Faul, Universität Kiel, Germany).

The participants of the study were asked about their age, gender and year of education as personal information and the data were recorded.

### Learning methods

Before the COVID-19 pandemic, the (preclinical) learning strategies in the dentistry academic study program at Suleyman Demirel University Faculty of Dentistry were practice-based student-centered active learning. In the first two years of the dentistry program, intense theoretical and practical training in basic medical sciences are provided, while specific dentistry courses are provided from the 3rd year. While the 1st and 2nd-year students practiced dentistry in preclinical laboratories, the 3rd-year students had the opportunity to observe the clinics. Fourth and 5th-year students spent half of the day treating patients in the clinic and the other half with theoretical education.

With the COVID-19 pandemic, profound changes had to be made in the education method of the denstistry program. The COVID-19 pandemic protocol mandated distance learning; dentistry education was switched completely to distance learning for all courses as of March 12, 2020. Education for 1st, 2nd, and 3rd year students, who are predominantly provided theoretical education, were offered online classes. Lectures, group discussions, case presentations, seminars assignments, and assessments were conducted using various online platforms (Adobe Connect, Microsoft Teams, Zoom, etc.). Video simulations of practical and laboratory lessons involving various psychomotor skills were provided. For 4th and 5th year students, face-to-face participation in the clinics was conducted with all necessary precautions.

### Questionnaire

The questionnaire was developed to evaluate the students' quality of life (QoL) and their psychosocial status during the process of distance learning. The World Health Organization Quality of Life Scale-Short Form (WHOQOL-BREF) was used to evaluate QoL of the students. The DASS-21 scale was used to evaluate anxiety, depression, and stress. The questionnaires were prepared in Google Forms program and sent to the students via e-mail and direct message. The first part of the questionnaire consisted of questions regarding the student's gender, age, and year of education.

#### WHOQOL BREF

WHOQOL-100 with 100 questions and WHOQOL-BREF with 26 questions had been selected as scales to determine QoL from pilot studies carried out in 15 centers worldwide. The WHOQOL-BREF scale consists of 26 questions, including one question on the general QoL and the other on perceived health satisfaction. There are four domains in the scale: physical, psychological, social relationships, and environmental. The answers to the questions are given as 1–5 points on a 5-point Likert scale, and the field scores are converted into points out of 100. The Turkish validity and reliability of the questionnaire were carried out by Eser et al. [16]. The 27th question was added to the Turkish version, and this question was included in the calculation of the perimeter score.

The questions were asked about the previous 15 days. The WHOQOL-BREF scale does not have a total score. A single quality of life score cannot be reached by adding the scores of all domains. Four field scores are calculated with this scale as follows:

- 1. *Physical health*: activities of daily living, need for medical care, fatigue, mobility, pain, sleep, rest, and work capacity.
- 2. *Psychological*: body image, positive and negative thoughts, self-confidence, religious and personal beliefs, thinking, learning, memory, and concentration.
- 3. *Social relationships*: assesses interpersonal relationships and social support.
- 4. *Environmental*: financial resources, physical security, accessibility and quality of health and social services, home environment, opportunities to access new knowledge and skills, physical environment (pollution, noise, traffic, climate), and transportation.

#### DASS-21

The second part of the questionnaire included 21 items from the short-form version of DASS. Lovibond and Lovibond developed DASS to assess the main symptoms of depression, anxiety, and stress. DASS showed satisfactory psychometric properties and is comparable to other reliable scales. The Turkish validity and reliability of the questionnaire were carried out by Yılmaz et al. The DASS-21 is a short-form version of the self-reported 42-item questionnaire and showed good to excellent internal consistency, adequate reliability, and construct validity.

DASS-21 includes three self-reported scales designed to measure negative emotional states of depression, anxiety, and stress. Each of the three scales contains seven items scored on a Likert scale of 0–3 (0: It does not suit me at all, 1: Applies to me to some extent or for some time, 2: Applies to me significantly or most of the time, 3: Applies to me a lot or most of the time). Depression, anxiety, and stress scores are calculated by summing the scores of the related items. Since DASS-21 is a short-form version of DASS (42 items), the final score of each subscale is multiplied by two and evaluated according to the severity index.

Data from both the QoL scale and the DASS-21 questionnaire were recorded for each student. The students were separated according to their year of education prior to the analysis of their data. According to the learning methods, the 1st, 2nd and 3rd year students who received online learning belonged to the distance learning group; 4th and 5th-year students who received clinical education and internships were included in the face-to-face/classroom learning group.

### Statistical analysis

The statistical analysis was performed using Statistical Package for Social Sciences software (SPSS version 20; IBM Corporation, New York, USA). Descriptive statistics (mean and standard deviation) were calculated to assess the quality of life and depression, anxiety, and stress levels of the study participants. Cronbach's alpha was used to measure the internal consistency and reliability of the questionnaire. The data obtained were compared between two different groups: classroom/face-to-face learning and distance/online learning. One-way ANOVA was used for comparison of students in the different years of education; post hoc LSD test was used for pairwise comparisons. Sample t-test was used to compare the two groups separated as classroom/face-to-face learning and distance/online learning. The level of statistical significance was at 0.05.

## Results

**Fig. 1:**
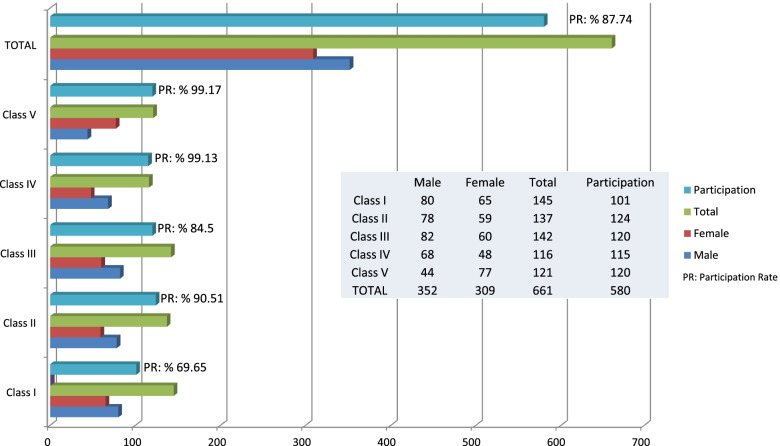
Distribution of students studying in our faculty according to classes and participation rates

Validated Turkish versions of the questionnaires were used in the current study. The questionnaire was sent to 641 of the 661 students studying at the faculty of dentistry. Twenty students were excluded as they were foreign nationals and therefore not adept at Turkish. In total, 601 of 641 students from all study years were invited to participate. The questionnaire was completed by 580 students, indicating a response rate of 87.74%. The demographic characteristics of the study participants are presented in Fig. 1. Cronbach's alpha values used to measure the internal consistency and reliability of the questionnaire were found to be high for both scales used in the study (QoL scale, α ≥ 0.948; DASS-21 scale, α ≥ 0.961).

According to results of the QoL scale, there was no significant difference between the in-class and distance education groups regarding general health, physical health, and psychology (*p* > 0.05) (Table 1). Additionally, no significant difference in social relationships could be identified between the in-class and distance education groups (*p* > 0.05). However, a significant difference between the QoL scores of the students at the 1st and 4th-year of education was identified (*p* < 0.05). 1st year students reported high scores in the environmental sub-unit, which contributed to the significant difference observed between the students at the different years of education (*p* < 0.05), although the groups were statistically similar according to the learning methods (*p* > 0.05) (Table 2).

**Table 1:** Statistical comparison of the scores of the QoL and DASS-21 scales according to their classes

	**Dental Students’ Classes**															
	1	2	3	4	5	**Post-Hoc Tests**										***p***
	**Mean ± SD**	**Mean ± SD**	**Mean ± SD**	**Mean ± SD**	**Mean ± SD**	1–2	1–3	1–4	1–5	2–3	2–4	2–5	3–4	3–5	4–5	
	**DASS 21**															
**Anxiety**	14.37 ± 6.55	13.97 ± 6.48	16.56 ± 7.04	15.74 ± 6.56	15.15 ± 6.52	NS	*	NS	NS	**	NS	NS	NS	NS	NS	**.040**
**Depression**	15.03 ± 8.25	14.67 ± 6.93	17.3 ± 9.01	15.79 ± 8.28	14.41 ± 8.35	NS	NS	NS	NS	*	NS	NS	NS	**	NS	**.038**
**Stress**	13.9 ± 6.31	12.5 ± 6.27	15.79 ± 7.98	15.23 ± 7.74	15.05 ± 6.75	NS	NS	NS	NS	**	**	*	NS	NS	NS	**.016**
	**WHOQOL BREF**															
**General Quality Of Life**	7.14 ± 1.59	7.03 ± 1.61	6.8 ± 1.71	6.86 ± 1.49	6.89 ± 1.49	NS	NS	NS	NS	NS	NS	NS	NS	NS	NS	**NS**
**Physical Health**	22.75 ± 3.94	23.24 ± 3.7	22.25 ± 3.98	21.75 ± 3.76	22.56 ± 3.41	NS	NS	NS	NS	NS	**	NS	NS	NS	NS	**NS**
**Psychological**	19.95 ± 3.22	20.56 ± 3.56	19.92 ± 3.96	19.52 ± 3.37	20.33 ± 3.51	NS	NS	NS	NS	NS	*	NS	NS	NS	NS	**NS**
**Social Relationships**	9.22 ± 2.79	10.5 ± 2.33	10.36 ± 2.62	9.49 ± 2.71	10.09 ± 2.54	**	**	NS	*	NS	**	NS	**	NS	NS	**.004**
**Environmental**	29.25 ± 5.42	28.37 ± 5.26	27.4 ± 5.44	27.11 ± 4.93	28.56 ± 5.58	NS	*	*	NS	NS	NS	NS	NS	NS	*	**.033**
**Total**	88.12 ± 13.78	90.21 ± 13.72	86.86 ± 14.43	84.15 ± 13.14	88.33 ± 13.69	NS	NS	NS	NS	NS	**	NS	NS	NS	*	**.037**

Abbreviations: *NS* Not significant, *p*, Results of One-way ANOVA test (Post Hoc (LSD) test), *SD* Standard Deviation; *P < .05; **P < .01; ***P < .001

**Table 2:** Statistical comparison of the scores of the QoL and DASS-21 scales according to learning methods

	Distance (Online) Learning	Classroom (Clinical) Learning	*p*
DASS 21	**Mean ± SD**	**Mean ± SD**	
Anxiety	15.46 ± 6.54	15.41 ± 6.88	.935
Depression	15.13 ± 8.32	16.12 ± 8.41	.170
Stress	15.14 ± 7.28	14.51 ± 7.34	.317
WHOQOL BREF	**Mean ± SD**	**Mean ± SD**	
General Quality Of Life	6.87 ± 1.49	6.93 ± 1.66	.644
Physical Health	22.14 ± 3.61	22.62 ± 3.91	.140
Psychological	19.91 ± 3.46	20.1 ± 3.71	.557
Social Relationships	9.79 ± 2.64	10.17 ± 2.62	.101
Environmental	27.81 ± 5.29	28.05 ± 5.42	.611
Total	86.26 ± 13.55	88.02 ± 14.13	.154

*P*: Results of Sample t-test; *SD* Standard Deviation; *NS* Not significant, *P* > 0.05

According to the results of the DASS-21 scale, the anxiety level in the 3rd year students (16.56 ± 7.04) was significantly higher than in the other years (*p* < 0.05) (Table 1). However, this difference was statistically similar when evaluated according to the learning methods (*p* > 0.05). Depression level in the 3rd year students was also found to be significantly higher (17.3 ± 9.01) compared to the other years (*p* < 0.05). This difference was again statistically similar when the two groups were compared according to the learning methods (*p* > 0.05) (Table 2). Stress level of the 2nd years students (12.5 ± 6.27) was statistically significantly different from the other years (*p* < 0.05). When examined according to learning methods, differences in stress levels did not reach statistical significance (*p* > 0.05).

## Discussion

Social distancing measures became necessary during the COVID-19 pandemic. Such measures, however, have a significant impact on practical training, such as dentistry training [17, 18]. Online education allows participation in distance learning events regardless of location [19]. Therefore, most universities launched online learning in the spring of 2020, in line with government guidelines in implementing measures to prevent the spread of the virus and contain the pandemic. Most students continued their education online after the spring break in 2020 [20].

The implementation of distance learning in dentistry education in low-middle-income countries is challenging due to reasons such as: (1) technology/infrastructure barriers, (2) institutional/educational barriers (3) student barriers. Due to these obstacles, distance education has caused considerable difficulties for both students and faculty members [10, 21, 22].

In the current study, we observed that the overall early experience of dental students in distance learning was better than expected. We think that our students showed higher satisfaction due to their previous experience in distance learning. Thus, a combination of traditional and e-learning may be suitable as an alternative for future dentistry education. The QoL scale results indicated no significant difference between the groups in general health, physical health, and psychology, irrespective of year or education and learing method (distance versus in-class). This is reflective of the high level of satifcation of the students with the distance education provided in our institution.

Dental students were under stress due to the COVID-19 pandemic, emanating from conflicts between returning to clinical courses, disruptions in clinical education and from the perceived risk of infection upon contact with patients. Unfortunately, there is no gold standard for education of dental students during a global pandemic. Although the increase in COVID-19 cases has somewhat slowed down, researchers predict it will take time to transition to 'normal’ education [20]. Therefore, there is a need to develop more flexible educational tools as well as curriculum with more online learning without the need for "face-to-face" contact. The early-stage results of our survey point to an opportunity to minimize the impact of the steps taken to manage the crisis on dental teaching and to be better prepared for similar disruptions in the future.

The current study was carried out during the social isolation period; therefore, an exacerbation of stressors that may have been considered minor in pre-pandemic times was observed, as also reported in other studies. These exacerbations were primarily observed during the transition from preclinical to clinical classes [23–28].

We observed no significant difference in social relationships score between the groups according to learning methods; however, a significant difference in the scores was observed according to the year of education. This difference was primarily due to lower scores observed with the 1st and 4th-year students. For 1^st^ year students, the low score can be attributed to the lack of opportunities to get to know the environment and their friends due to the transition to online education. For 4th year students, the low score could have resulted from socialization and anxiety parameters of the students as they were expected to transit towards starting treatment of patients as a trainee clinician in the middle of a global pandemic.

Lack of social support is known to harm the mental wellbeing of healthcare professionals. High stress levels in healthcare workers during the COVID-19 pandemic were most likely due to the fear of transmitting the virus to loved ones and anticipating what might happen to them, which has been reported to impose a significant psychosocial burden [29, 30]. Independent of the current global pandemic, providing social support to healthcare professionals to improve their self-confidence in managing work-related stress is likely to yield better outcomes.

Clinical training during undergraduate education is often patient-centered. The sudden closure and cancellation of all clinical activities of certain medical training institutions during the COVID-19 pandemic was due to the unpreparedness of these institutions [31]. This had adverse psychological effects among future medical students, including being unable to focus during self-study and preparing for college-leaving exams to become more self-confident and more competent doctors in the future, along with deterioration in self-confidence and independence. A recent survey of 32 medical schools in the UK found that the COVID-19 pandemic had significantly impacted willingness to work and self-confidence of students. Such an event is therefore likely to affect the transition from student to professional life [7, 32].

In the environment sub-unit, the high scores obtained with the 1st year students generated a significant difference between the students at different years of education while the groups separated according to learning methods did not show a significant difference in the scores. The environment sub-unit score is expected to feature crucially among 1^st^ year students who will have missed the opportunity to get to know the environment, university, and their friends owing to online learning.

A Swedish population study reported an association between varying stress levels and mental health such that depression was associated with high-level stress, while anxiety was observed as a result of low- and moderate-level stress [33]. Anxiety and depression among dentistry students are not as frequently investigated as stress, despite the close relationship between them. In previous studies, stress was assessed solely with the Dental Environmental Stress (DES) Scale. It would be helpful to examine depression, anxiety, as well as stress with a single scale rather than multiple assessments. The Depression, Anxiety, and Stress Scale (DASS-21) is one of the best tools to measure all three dimensions of these psychological states in a single, short and comprehensive manner. To our knowledge, DASS-21 scale has not been applied to measure the psychological states of dentistry students during the COVID-19 pandemic [15].

Naidu et al. reported a remarkable increase in the intensities of stress levels during the transition from preclinical classes to clinical classes, similar to the results of the current study [34]. The stress level associated with clinical activities is generally high for students in their third year – significantly higher than the second year and only slightly lower than the fifth (final) year. A similar exacerbation of stress associated with transitioning from preclinical to clinical activities was reported by Hayes et al. [26]. The high scores in stress and anxiety parameters in 2nd year students observed in the current study was most likely due to the transition of practical classes to online lectures leaving the students with inadequate experience in hands-on practical training.

In many countries, dental students are selected from individuals with the highest grades and performance indicators after a grueling and high pressured selection process. This pressure continues throughout dental education. The need for students to master the technical skills and new theoretical knowledge required to successfully complete dental education has increased the stress on students [15]. Moreover, the need for students to master technical skills and new theoretical knowledge has increased the stress on students even more. Schmitter et al. suggested that dental education was even more stressful than medical education [12, 15]. In studies conducted prior to the COVID-19 pandemic, stress associated with dentistry students resulted from insufficient allocation of training at the clinics especially for 3^rd^ year students, stress of meeting clinical commitments, lack of confidence in the student's own clinical decision-making, and differences in opinions with the clinical staff about treatment. Patient management and acquisition and perfection of manual dexterity, however, were associated with lower stress levels [27]. Being selected for a highly demanding training and profession does not mean that students are more resilient to stress. The COVID-19 pandemic has brought about well-documented stress crises in the population [35, 36]. Such a level of stress can make a considerable negative impact on the stamina and strength of future doctors in dealing with crises such as difficult patients and complicated surgeries [7].

The limitations of the study include the fact that the surveys were conducted only in a single educational institution. The pandemic was not planned, and it still continues, so that it is not possible to apply a survey to the same student group before and after the pandemic. It can be predicted that this pandemic will bring significant physical and psychological changes in future dentists, both in their private and professional lives. Future research should focus on the long-term psychological impact of COVID-19 globally and what can be done to counter its many consequences. Further studies could focus on a longitudinal evaluation of the psychosocial situations of the same student group in different classes throughout their education, as a multi-center study, with larger sample sizes.

## Conclusion

Anxiety, stress and factors affecting the quality of life negatively affected dental students who received distance learning, although the difference did not reach statistical significance when compared to students who received in-class learning. 3rd year students who were scheduled to receive practical clinical training showed high levels of stress, anxiety and depression due to the pandemic/quarantine and distance learning. Dental students are individuals who are mostly young, resilient, less prone to anxiety, and have an active social life. But the COVID-19 pandemic continues to change our lives, our health, with great uncertainty about the long-term impact, and students are at risk of poor quality of life due to the experience of social isolation.

## Data Availability

The datasets used and/or analyzed during the current study are available from the corresponding author on reasonable request.

## Abbreviations

WHO: World Health Organization
QoL: Quality of Life
WHOQOL-BREF: The World Health Organization Quality of Life Scale-Short Form
DES: Dental Environmental Stress Scale
DASS-21: The Depression, Anxiety and Stress Scale
SPSS: Statistical Package for Social Sciences software
